# Dietary Protein Intake and Overall Diet Quality are Associated with Handgrip Strength in African American and White Adults

**DOI:** 10.1007/s12603-018-1006-8

**Published:** 2018-02-22

**Authors:** Marie Fanelli Kuczmarski, R. T. Pohlig, E. Stave Shupe, A. B. Zonderman, M. K. Evans

**Affiliations:** 1 University of Delaware, Department of Behavioral Health and Nutrition, 206C McDowell Hall, Newark, DE 19716 USA; 2 University of Delaware, College of Health Sciences, STAR, Newark, DE 19716 USA; 3 Laboratory of Epidemiology and Population Sciences, National Institute on Aging, NIH, 251 Bayview Blvd. Suite 100, Baltimore, MD 21224-6825 USA

**Keywords:** Handgrip strength, protein, diet quality, African American, body mass index

## Abstract

**Objective:**

To determine the association of handgrip strength (HS) with protein intake, diet quality, and nutritional and cardiovascular biomarkers in African American and White adults.

**Design:**

Cross-sectional wave 3 (2009–2013) of the cohort Healthy Aging in Neighborhoods of Diversity across the Life Span (HANDLS) study.

**Participants:**

Socioeconomically diverse urban population of 2,468 persons aged 33 to 71 years.

**Measurements:**

Socio-demographic correlates, dietary intakes and biomarkers, HS, physical performance measures were collected. HS was measured using a dynamometer with the dominant hand. Functional measures included chair, tandem, and single leg stands. Two 24-hour recalls were collected using the US Department of Agriculture Automated Multiple Pass Method. The total protein intake and diet quality, evaluated by adherence to the DASH eating plan and Healthy Eating Index-2010, were calculated. Biomarkers included nutritional anemia, and serum levels of albumin, cholesterol, magnesium, and glucose.

**Results:**

The mean ±SE age of the sample was 52.3±0.2 years. Approximately 61% were African American and 57% were women. The mean ±SE HS of women was 29.1±0.2kg and for men was 45.9±0.4 kg. Protein, gm, per kg body weight for the women was 0.94±0.02 compared to 1.16 ±0.02 for men. After adjusting for socio-demographic factors, hypertension, and diabetes, HS/BMI ratio was significantly associated with protein intake per kg body weight (p<0.001) and diet quality, assessed by either the DASH adherence (p=0.009) or Health Eating Index-2010 (p=0.031) scores. For both men and women, participants in the upper tertile of HS maintained a single leg and tandem stances longer and completed 5 and 10 chair stands in shorter time compared to individuals in the lower HS tertile. Of the nutritional status indicators, the percent of men in the upper HS tertile with low serum magnesium and albumin, was significantly lower than those in the lower HS tertile [magnesium,7.4% vs 16.1%; albumin, 0.4% vs 4.5%]. The only difference observed for women was a lower percent of diabetes (14.4% for the upper HS tertile compared to 20.5% for the lower HS tertile.

**Conclusions:**

The findings confirm the role of protein and a healthful diet in the maintenance of muscle strength. In this community sample, HS was significantly associated with other physical performance measures but did not appear to be strongly associated with indicators of nutritional risk. These findings support the use of HS as a proxy for functional status and indicate the need for research to explore its role as a predictor of nutritional risk.

## Introduction

Universally, handgrip strength (HS), a muscle strength measurement (1), declines with age and predicts future disability and mortality (2-5). It is considered a reliable tool for assessing nutritional status across income groups in clinical practice (6, 7). HS is one of the six characteristics included in the recommendations to diagnose adult malnutrition (8). The use of clinically relevant HS indices to identify older adults who are at risk for functional impairment, weakness and low muscle mass has also been recommended (1, 9). Evidence exists that muscle strength per body mass index (BMI) would be an appropriate relative strength index in clinical settings (1, 10-12). However, research in community settings and the association of this index with protein intake and diet quality has not been fully investigated (13-16).

Cross-sectional studies have documented that a healthful diet is associated with better muscle strength and physical performance (5). In northern European women, high adherence to a Mediterranean eating pattern was positively associated with indices of skeletal muscle mass and function (17). Dietary protein intake is also associated with maintenance of muscle mass and physical function with aging. Women who participated in the OSTPRE- Fracture Prevention Study and consumed high protein intakes (>1.2gm/kg) had less decline in HS adjusted for body mass over 3 years and had better performance in HS/body mass, single leg stand, and chair stand at baseline compared to women who consumed moderate (0.81-1.19gm/kg) and low (<0.8gm/kg) intakes of protein (18). Furthermore, McLean and colleagues found that higher intakes of total and animal protein expressed as gram per kg body weight were protective against loss of HS in men and women aged 60 years and older from the Framingham Offspring Cohort, a primarily white, middle-class sample (16). However, the relationship of dietary protein intake and diet quality to muscle strength across races remains unclear.

Muscle strength may also play a role in cardiometabolic disease, and HS has been proposed as a potential marker for detecting undiagnosed disease among adults at normal weight (19). Among healthy weight adults with no history of cardiovascular disease examined in NHANES 2011-12, HS was lower in individuals with diagnosed and undiagnosed hypertension and diabetes compared to individuals without hypertension or diabetes (19). An inverse association between dietary magnesium intake and cardiovascular risk and diabetes was also found in prospective cohort studies (20-22). The role of magnesium in muscle function is widely recognized, emphasizing the importance of diet (23, 24).

HS is dependent on many factors such as sex, age, and race (25-27). Men have higher HS than women of similar ages (28-30). Peak HS occurs in young adulthood followed by accelerated decline beginning after 40 years (28, 31, 32). HS also differs among African Americans and Whites. HS of African American women is greater than that of White women, regardless of income status (26). However, this finding was inconsistent for men (26). The usefulness of HS for nutritional screening in a community setting with racially diverse populations of similar ages has not been extensively studied.

A comprehensive review of the literature did not reveal any studies which explored the association of HS/BMI ratio with protein intake and diet quality. Thus the primary objective of this study was to determine the association of HS/BMI ratio with protein intake and diet quality adjusting for demographic and cardiovascular risk factors in a racially diverse urban population. The second objective was to explore the relationship of HS with selected nutritional status indicators and physical performance measures to evaluate the usefulness in community-based assessments of nutritional risk.

## Methods

### Healthy Aging in Neighborhoods of Diversity across the Life Span (HANDLS) Study Background

The HANDLS study, a 20-year prospective study initiated in 2004, has been described in detail elsewhere (33). Participants were drawn from 13 pre-determined Baltimore neighborhoods, yielding a representative factorial cross of four factors: age (30 to 64 years), sex (men and women), race [African Americans (AA) and Whites (W)], and income (self-reported household income <125% and ≥125% of the 2004 Health and Human Services poverty guidelines) (34), with approximately equal numbers of subjects per factorial cell.

There were two interview sessions in the Wave 3 HANDLS study, 2009–2013. The first session was completed on the Mobile Research Vehicles (MRV) located in participants’ neighborhoods or homes. This session consisted of a medical history, physical performance assessments, physical examination, cognitive evaluation, laboratory measures, and the first 24-hour dietary recall. The second session was done approximately 7–10 days later and consisted of the second 24-hour dietary recall and dietary supplement questionnaire completed over the telephone. Study protocol was approved by National Institute of Environmental Health Sciences IRB and the IRB at the University of Delaware. All HANDLS participants provided written informed consent following their access to a protocol booklet in layman’s terms and a video describing all procedures. They were compensated monetarily.

### Sample

In baseline HANDLS study a total of 3,720 AA and W participants were examined. Of these participants, 2,468 were reexamined in Wave 3. Only 1,787 individuals [1,009 women, 776 men] completed HS measures. Of those with HS measures, 1,714 persons [984 women, 730 men] completed two days of 24-hour dietary recalls.

### Physical Performance Measures

HS was assessed by trained technicians using the Jamar Hydraulic Hand Dynamometer (Patterson Medical Holdings Inc., Bolingbrook, IL)(35). The participants were in a seated position with the elbow of the tested side resting on a table at approximately 160°. The hand dynamometer registers the maximum kilograms of force per trial, where two trials were performed for both the right and left hands with a 15–20 second rest between trials. If the participant reported surgery within the past three months or if they had pain and/or arthritis that would impede their ability to successfully complete the handgrip test, the test was not performed. The maximum force of the dominate hand was used for this study. For those who reported that they were ambidextrous, the right-hand measure was used.

Physical performance was measured by a modified short physical performance battery (SPPB) evaluation which included tests of standing balance tandem stand, chair stands, and single leg stands (36). Only one full tandem leg stand for 30 seconds was performed, while the chair stands were increased from 5 to 10 repetitions. The single leg stand, the surrogate for the gait test in the HANDLS study, was performed three times with maximum time of 30 seconds per trial.

### Dietary Method

The United States Department of Agriculture (USDA) computerized Automated Multiple Pass Method was used to collect both 24-hour dietary recalls (37). An illustrated Food Model Booklet, measuring cups, spoons, and ruler were used to assist participants in estimating accurate quantities of foods and beverages consumed. Both recalls were administered by trained interviewers. Dietary recalls were coded using Survey Net, matching foods consumed with 8-digit codes in the Food and Nutrient Database for Dietary Studies version 5.0 (38).

### Diet quality measures

The score for Dietary Approaches to Stop Hypertension (DASH) diet adherence was determined for each participant using the formula reported by Mellen et al (39). These researchers identified DASH goals for eight target nutrients, namely total fat, saturated fat, protein, fiber, cholesterol, calcium, magnesium, and potassium. Additionally, sodium was included as a target nutrient even though dietary sodium was held constant in the original DASH study. Micronutrient goals were normalized to 1000 kcal. The total DASH score was generated by the sum of all nutrient targets met. If the participant achieved the DASH target for a nutrient a value of 1 was assigned, and if the intermediate target for a nutrient was achieved a value of 0.5 was assigned. Zero was assigned if neither target was met. Individuals meeting approximately half of the DASH targets (DASH score=4.5) were considered DASH adherent (39).

Food-based diet quality was also evaluated with the Health Eating Index (HEI)-2010. The National Cancer Institute’s Applied Research Web site provided the basic steps for calculating the HEI-2010 component and total scores and statistical code for 24-hour recalls (40). A detailed description of the procedure used for this study is available on the HANDLS website (41). Component and total HEI-2010 scores were calculated for each recall day and were averaged to obtain the mean for both days combined.

### Anthropometric, Clinic, and Blood Measures

BMI (kg/m2) was calculated from measured weight and height. Weight was obtained using a calibrated Med-weigh, model 2500 digital scale, and height was measured with the participant’s heels and back against a height meter supplied by Novel Products, Inc.

Fasting venous blood specimens were collected from participants during their MRV visit and analyzed at the Nichols Institute of Quest Diagnostics, Inc. (Chantilly, VA, USA). Fasting blood results utilized for the present study included serum measures of albumin (g/L), magnesium (mg/dL), iron (mcg/dL), folate (ng/mL), B12 (pg/mL), ferritin (ng/mL), total iron binding capacity (TIBC)(mcg/dL), total cholesterol (mg/ dL), and hemoglobin (g/dL) and glucose (mg/dL). Serum albumin, magnesium, iron, and total iron binding capacity and glucose were measured by the standard clinical laboratory spectrophotometric assay. Serum ferritin was measured using a standard chemiluminescence immunoassay. Serum folate and vitamin B12 were measured using enzyme immunoassay. Total serum cholesterol was assessed using a spectrophotometer (Olympus 5400, Olympus, Melville, NY, USA). Highsensitivity CRP levels were assessed by the nephelometric method utilizing latex particles coated with CRP monoclonal antibodies.

To diagnose nutritional anemia, participants were first categorized by presence of anemia defined by a hemoglobin level less than 13 g/dL in men and less than 12 g/dL in women (42). Then, among those with anemia, participants with nutritional anemia due to inadequate iron, folate, and/or Vitamin B12 were identified. Nutritional anemia was defined as MCV ≤ 95 *μ*m3 accompanied by low ferritin levels (≤ 30 ng/mL) or MCV ≤ 95 *μ*m3 with normal ferritin levels (31-99 ng/mL) and low transferrin saturation (FeSat)(<16%) (FeSat = serum iron/TIBC)(43). Criteria used to identify anemia due to serum folate was <4 ng/mL and vitamin B12 was <200 pg/mL (44).

Several biomarkers were used to assess early signs of malnutrition. Cholesterol levels <160 mg/dL (45), albumin <3.5 mg/dL, and magnesium <1.7 mg/dL (46) were used to define inadequate levels of these biomarkers.

Hypertension was defined as systolic blood pressure (SBP ≥140 mm Hg), diastolic blood pressure (DBP) ≥90 mm Hg, a history of blood pressure medication use, or a self-report of diagnosed hypertension (45). SBP and DBP were assessed with the participant in a seated position following a 5-min rest. One measure was obtained on each arm and then those measures were averaged. Prediabetes and diabetes mellitus were defined as fasting glucose of 100–125 and ≥126 mg/dL (47), respectively, a history of medication use, or a self-reported diagnosis.

### Statistical Analysis

Means and standard errors for continuous variables and proportion of participants for relevant categorical variables were calculated. Analysis of variance (ANOVA) was used to compare demographic and life-style factors, diet quality, HS and physical performance measures, across age categories (33–59 years, 60–71 years), and p-values were adjusted for multiple comparisons of continuous variables using the Bonferroni test. For sample characteristics categorical data, χ2 tests were used. Statistical significance was established at P<0.05. All statistical analyses were performed with IBM SPSS Statistics for Windows v23.

Sex and race specific criteria for cut points were used to define the tertiles for HS. One- way ANOVA was used to compare physical performance measures across tertiles. The number of people unable to perform each measure was also tallied. In addition, the HS cutpoints published by Alley et al (12) were used to determine the number of persons with clinically relevant weakness. Weakness was associated with mobility impairment, defined as gait speed less than 0.8m/s (12). For comparisons of the proportion of the sample at nutritional risk, χ2 tests were used.

Sequential multiple regression models were used to test if diet quality and protein intake per kg body weight predicted HS/BMI ratio. In the first block were covariates and included age, sex, race, income, cigarette smoking status, diabetes, and hypertension. The second block contained protein (g) per kg body weight while the third block contained DASH diet score. The last block contained two-way interactions, specifically sex x race, sex x income, and race x income. Blocks in sequential regression refer to predictors that are entered simultaneously. Entering predictors in blocks allows for testing if the addition of multiple predictors simultaneously significantly improves the model.

**Table 1:**
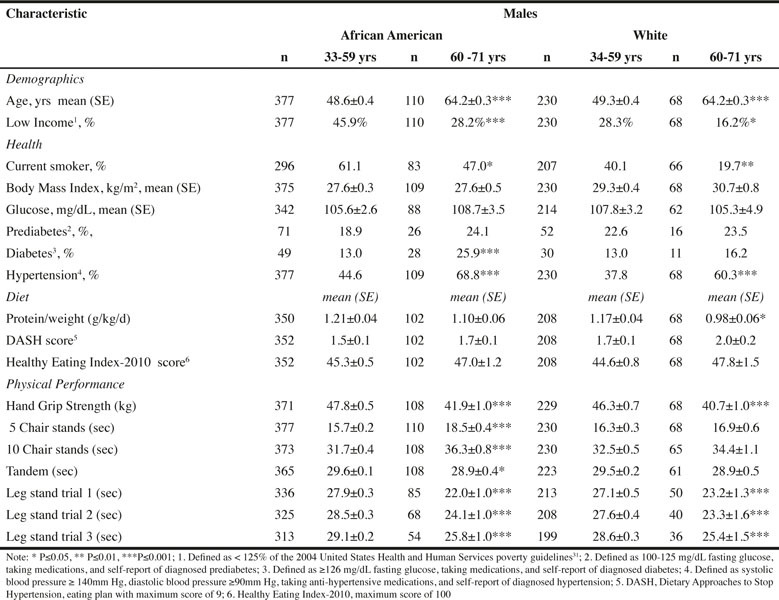
Characteristics of Male Participants in Healthy Aging in Neighborhoods of Diversity across the Life Span (HANDLS) Study by Age within Race Categories

Separate regression analyses were also performed with HEI- 2010, the results were the same as those with DASH (data not shown).

## Results

### Sample Characteristics

Approximately 23% of men and 24% of women were 60–71 years of age. The proportion of the population who currently smoked was significantly higher for the 33–59 years age category for all race-sex groups (Tables 1 and 2). Mean glucose was similar across age for all race-sex groups (Tables 1 and 2). Although the percent of individuals with prediabetes did not differ across age for either sex or race, diabetes was more prevalent among AA men and women and W women 60–71 years compared to women less than 60 years (Tables 1 and 2).

BMI was not significantly different within either sex by race for age category (Tables 1 and 2). The percentage of the population with incomes less than 125% of poverty guidelines was lower for both AA men and all women aged 60–71 years compared to their younger counterparts (Tables 1 and 2). The only significant differences in protein intake per kg body weight were found for W men, with the older age group consuming less protein per kg body weight compared to the younger age group. With respect to diet quality, no differences were found for men across age groups. For AA women, the mean DASH and HEI-2010 scores were higher for the older compared to younger age group. For W women, HEI-2010 scores, but not DASH scores were higher for the older compared to younger age group.

**Table 2:**
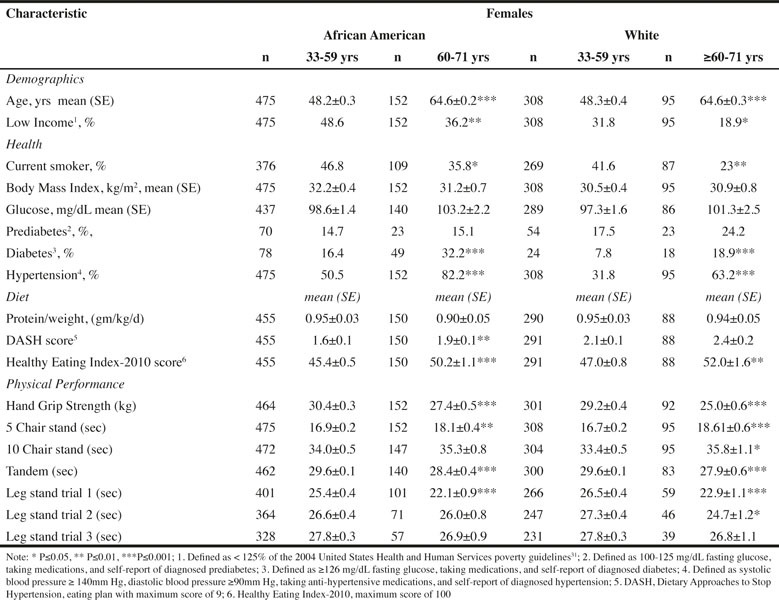
Characteristics of Female Participants in Healthy Aging in Neighborhoods of Diversity across the Life Span (HANDLS) Study by Age within Race Categories

### Handgrip Strength and Physical Performance Measures

Mean HS, and time to hold the single leg (first trial only) and tandem stands were significantly less, while time in seconds to complete 5 chair stands was significantly longer for women aged 60–71 years compared to those women less than 60 years (Table 2). The only significant difference in the second and third trials of the single leg stand was found for W women for the second trial. In addition, the time to complete 10 chair stands was significantly longer for the older compared to younger W women (Table 2). Comparable results were observed for men with these exceptions - time to complete 5 or 10 chair stands did not differ for W men; time to complete 5 or 10 chair stands was significantly longer for older AA men; and significant differences were found for all trials of the single leg stand, with the older group holding the stance for less time (Table 1).

As presented in Tables 3 and 4, the upper tertile of HS was associated with significantly better physical performance. Using the total sample of men or women, the time to complete 5 or 10 chair stands was significantly less for those participants in the upper tertile compared to the lower tertile of HS. With respect to the tandem stand and the first two single leg stands, persons in the upper tertile for HS held the stance for a significantly longer time than persons in the lower tertile for HS. With each subsequent single leg stand the number of persons unable to complete the trial increased (Table 3 and 4). None of the individuals in the upper HS tertile experienced weakness or intermediate mobility impairment.

Unlike the findings for the W men who had no significant difference in physical performance, the AA men in the upper HS tertile had significantly better physical performance for all measures compared to men in the lower tertile (Table 3). Among women, both W and AA in upper HS tertile had better physical performance with respect to 5 chair stands and the tandem and first single leg stands compared to women in the lower tertile (Table 4).

**Table 3:**
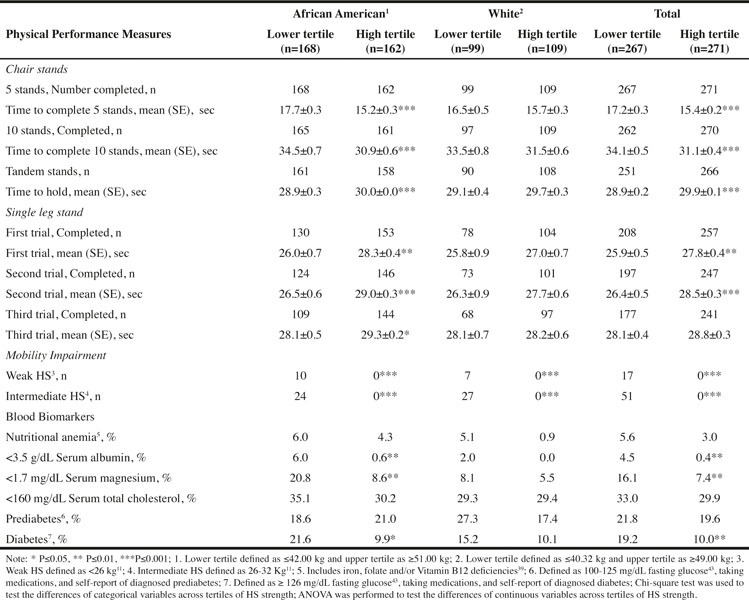
Physical Performance and Nutritional Status Biomarkers categorized by Hand Grip Strength for Males

### Biomarkers of Nutrition Status

There were some significant differences in the percentage of people with blood markers suggesting inadequate nutritional status between individuals in the lower compared to the upper tertile of HS. For the total sample of men, these biomarkers include low serum magnesium and albumin, and presence of diabetes (Table 3). As expected, the percentage was higher in the lower compared to the upper HS tertile. Significant differences in these 3 biomarkers were also observed for the AA but not the W men (Table 3). For the total sample of women and W women, the presence of diabetes was significantly higher in the lower compared to the upper HS tertile (Table 4).

The AA appeared to be at greater nutrition risk compared to the W. For instance, approximately 20% of AA men and women in the lower HS tertile had low serum magnesium concentrations compared to <10% of the W men and women. Mean c-Reactive Protein (cRP) was also calculated for the lower and upper tertiles for each sex-race group and no significant differences were found (data not shown).

**Table 4:**
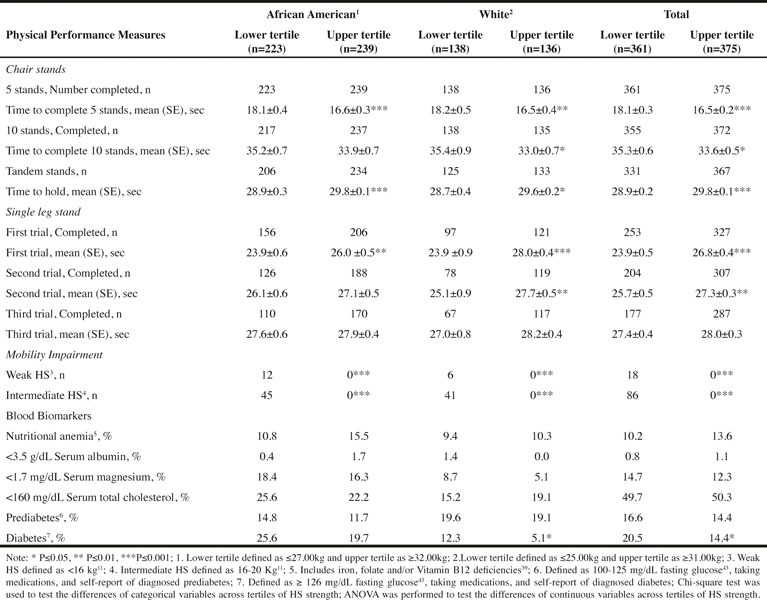
Physical Performance and Nutritional Status Biomarkers categorized by Low and Upper Tertiles of Hand Grip Strength for Females

### Variables Associated with Handgrip Strength

As shown in Table 5, after adjusting for age, sex, race, income, smoking, diabetes, and hypertension, protein per kg body weight was positively associated (P < 0.001) with HS/ BMI ratio. Using the same model, overall diet quality, as measured by adherence to the DASH eating plan, was tested and found to be positively associated (P < 0.009). Although the change in R2 was low, it was significant with the addition of the dietary variables. Amongst the covariates, being male, a nonsmoker, and not diabetic or hypertensive were associated with a higher HS/BMI ratio. Race and income were not associated with HS/BMI. Three interactions were tested (sex x race, sex x income, race x income), only the sex x race interaction was significant (P = 0.002). The overall R2 of the model was 0.566 (Table 5).

## Discussion

With increasing longevity, the preservation of muscle strength and quality are crucial for maintaining independence. The literature provides evidence that dietary protein intake, as part of an overall healthful diet, and physical activity can help protect against age-related muscle loss and functional decline (17, 18, 48-53). We are the first to report that relative HS, specifically HS/BMI ratio, was significantly associated with higher intakes of protein per kg body weight intake and better compliance to the DASH eating plan, adjusting for demographic factors, diabetes, and hypertension.

**Table 5:**
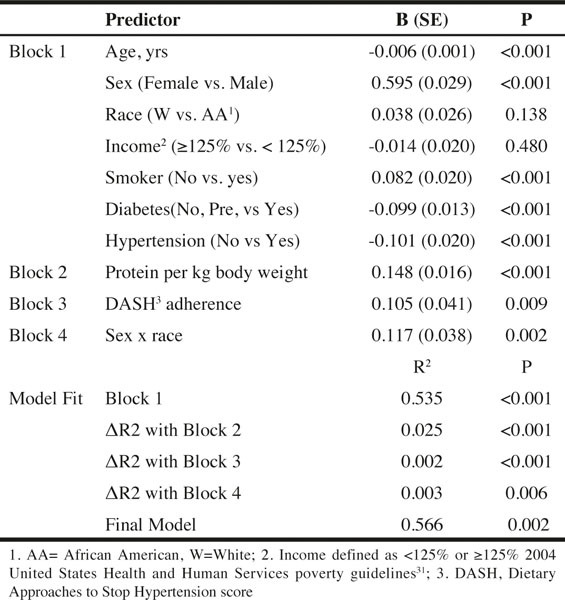
Hand Grip Strength per Body Mass Index as predicted by Protein Intake and Adherence to DASH Eating Pattern and Selected Sociodemographic Predictors: Regression model

Adherence to Mediterranean diet or DASH eating patterns, which are rich in antioxidant nutrients, such as magnesium and vitamins C and E, can lower inflammatory markers. Chronic low-grade inflammation and oxidative stress can trigger catabolism and increase protein turnover in skeletal muscle, reducing strength (54-56), as well as increase formation of reactive oxygen species resulting in an overload of the antioxidant defense system (57). Adherence to the DASH eating pattern was significantly associated with relative HS in this study. However, the association of adherence to Mediterranean diet with HS was only found significant in unadjusted analyses by Kelaiditi and colleagues (17). The researchers explained this lack of association by the fact that age was a strong determinant of HS in their cross-sectional studies (17). However, the differences might reflect the use of relative HS rather than absolute HS.

Similar to the findings of other researchers, the mean HS of the HANDLS study population was less for Whites compared to AA (26) and for women compared to men (31, 58). The mean and median HS of the HANDLS study participants were considerably lower than the mean and median HS reported for the National Health and Nutrition Examination Survey, 2011-12, which represents a national US sample (30). These reference values were categorized by age and sex for all races. The difference may be partially attributed to the different dynamometer model (59), since statistically significant differences in HS has been reported between the Jamar and Smedley dynamometers (60). Another possible explanation is that the HANDLS study participants are weaker but lean body measures would be needed to confirm this difference. HS measurements should be interpreted using ethnic/region specific reference ranges since HS values can vary not only due to dynamometer models used but also the calibration of these instruments, age categories, and differences in ethnicities, geographic regions, and physical activity levels (31, 32, 61). It is likely that the differences in HS also reflect variations in dietary patterns.

It is widely recognized that muscle strength declines (31, 32) while BMI increases with age (62). As anticipated the mean HS of the HANDLS study participants at Wave 3 was less than their reported values in the baseline phase of the study (26). Evidence exists that HS is positively associated with BMI, however this association may be less pronounced in obese individuals compared to individuals of other BMI categories (31, 61). The mean BMI of the HANDLS study participants indicates that overweightness and obesity are prevalent in this population. Some researchers have reported that HS/BMI ratio is the best predictor of mobility impairment for women (12). Among the HANDLS study sample, weakness was present in approximately 27% of persons within the lower HS tertile. Obesity combined with muscle weakness has been associated with a 3.9 fold greater risk of developing mobility limitation (63).

In this urban population, HS was significantly associated with other physical performance measures similar to findings of Stevens and colleagues (64). However, HS did not appear to be strongly associated with indicators of nutritional risk. These findings differ from those reported in clinical settings where HS can be a sensitive method for the diagnosis of malnutrition (3, 65). Yet they are consistent with the results of Springstroh and colleagues who found that HS was weakly associated with nutritional risk in community-dwelling older adults (66). While low hemoglobin has been reported to contribute to low HS independent of inflammatory markers and age (67), in our sample there were no differences in the percent of the population with nutritional anemia when comparing the lower to upper HS tertile. A single measure of HS may be appropriate for nutrition screening while HS variation over time may be better for nutritional status assessment in community-dwelling populations. Regardless, early identification of older adults at malnutrition risk is beneficial for the initiation of nutritional interventions (68).

The association of HS with nutritional biomarkers appeared to be stronger for men than women but the findings were inconsistent across the sexes by race. For example, low levels of serum albumin and magnesium, as well as presence of diabetes, were significantly more prevalent for men in the lower, compared to the upper HS tertile, while only the presence of diabetes was significant for women. The observation that lower HS was associated with greater prevalence of diabetes is consistent with the results of Mainous and colleagues (19).

As with any study there are strengths and limitations. The strengths of the study include the use of two 24-hour recalls for the evaluation of adherence to the DASH eating pattern, inclusion of a racially diverse independent population younger than 60 years of age, and confirmation of the regression findings using two diet quality indices. Limitations include the lack of persons over 71 years of age, of dual-energy X-ray absorptiometry data and physical activity measures, and the small number of nutritional biomarkers.

In conclusion, the findings support the recognized association between protein intake, healthful diet and HS. There is evidence that higher levels of muscle strength in older adults are seen with protein intakes ≥ 1.2g/kg body weight / day (51, 69). To achieve this level, protein enrichment with familiar foods can be an effective strategy (70). The dietary protein content for an optimal diet is currently under review with a focus on not only the total amount but also the amino acid content, quality, digestibility and daily protein distribution (71, 72). The results also support the use of HS as a proxy for functional status when assessing nutritional status risk in community settings. However, HS was not consistently associated with nutritional status indicators used in this study. Given the ease and inexpensive costs of obtaining HS, there is a need for research to further explore its role with other markers of nutritional risk in noninstitutionalized populations.

*Ethical Standards Disclosure:* This study was conducted according to the guidelines laid down in the Declaration of Helsinki and the study protocol was approved by Institutional Review Boards at National Institute of Environmental Health Science and the University of Delaware. Written informed consent was obtained from all subjects.

*Statement of Potential Conflict of Interest*: No potential conflict of interest was reported by the authors.

*Financial disclosure:* All authors have no financial disclosures.

*Acknowledgements:* This work is supported by the Intramural Research Program, National Institute on Aging, National Institutes of Health, grant Z01-AG000194.
